# Gene set-based identification of two immune subtypes of diffuse large B cell lymphoma for guiding immune checkpoint blocking therapy

**DOI:** 10.3389/fgene.2022.1000460

**Published:** 2022-10-07

**Authors:** Zhe Li, Ying Duan, Qing Ke, Mingyue Wang, Hong Cen, Xiaodong Zhu

**Affiliations:** ^1^ Department of Haematology/Oncology and Paediatric Oncology, Guangxi Medical University Cancer Hospital, Nanning, China; ^2^ Department of Oncology, Wuming Hospital of Guangxi Medical University, Nanning, China; ^3^ Department of Radiation Oncology, Guangxi Medical University Cancer Hospital, Nanning, China

**Keywords:** diffuse large B cell lymphoma, immune subtype, immune microenvironment, immune checkpoint blocking therapy, gene set-based identification

## Abstract

**Background:** Diffuse large B cell lymphoma (DLBCL) is the most common lymphoma in adults. Tumour microenvironment is closely related to tumour prognosis and immune checkpoint blocking therapy (ICBT). This study aimed to investigate the immunological and prognostic characteristics of the tumour microenvironment (TME), as well as the regulatory mechanisms.

**Methods:** Gene expression profiles and clinical data of patients with DLBCL were obtained from GEO database. ESTIMATE, CIBERSORT, and ssGSEA analyses were used to explore microenvironment characteristics and regulatory mechanism of the immune subtypes, which were identified by consistent clustering. The differences in enriched pathways were showed by GSEA. Hub genes associated with CD8^+^ T cells, which were identified by WCGNA, were exhibited biological functions through GO and KEGG. Immune-related gene scores (IRGSs) based on hub genes were used to evaluate the prediction of immune subtypes and ICBT, and retrospective analysis was used for validation. Finally, prognostic genes were screened to construct risk models.

**Results:** Consensus clustering divided patients with DLBCL into two subtypes with significant heterogeneities in prognosis and immune microenvironment. Low immune infiltration was associated with poor prognosis. Subtype C1 with high immune infiltration was enriched in multiple immune pathways. We observed that two common mutated genes (*B2M* and *EZH2*) in DLBCL were closely related to MHC-I and microenvironment. Our further analysis manifested that MYD88^L265P^ may be the main cause of TLR signalling pathway activation in subtype C1. Hub genes (*SH2D1A*, *CD8A*, *GBP2*, *ITK*, *CD3D*, *RORA*, *IL1R2*, *CD28*, *CD247*, *CD3G*, *PRKCQ*, *CXCR6*, and *CD3E*) in relation with CD8^+^ T cells were used to establish IRGS, which was proved an accurate predictor of immune subtypes, and patients in high-IRGS group were more likely to benefit from ICBT. Retrospective analysis showed that absolute lymphocyte count (ALC) was higher in the group that responded to the PD-1 inhibitor. Finally, the risk model was constructed based on two genes (*CD3G* and *CD3D*), and the low-risk group showed better prognosis.

**Conclusion:** DLBCL immune classifications with highly heterogeneity are a powerful predictor of prognosis and ICBT. The IRGS is proved to be a reliable tool to distinguish immune subtypes as a substitute for gene expression profile.

## Introduction

Diffuse large B cell lymphoma (DLBCL) accounts for 30–58% of all diagnosed cases of non-Hodgkin lymphoma, with an annual incidence of 1–5/10,000 (Li and Young, 2018). Further, DLBCL is a complex aggressive cancer with heterogeneous phenotypic, clinical, and molecular manifestations (Wright et al., 2003; Abramson and Shipp, 2005; Pan et al., 2015). Currently, R-CHOP (rituximab, cyclophosphamide, doxorubicin, vincristine, and prednisone) is the standard first-line treatment (Coiffier et al., 2002), and although cure rates have improved to 60–70%, nearly 40% of patients still present with refractory or relapsed disease (Younes, 2015). Reliable prognostic stratification and first-line/salvage treatment strategies still fail to meet clinical requirements, and the search for novel prognostic and treatment predictors has become an urgent issue.

Studies have confirmed that the tumour microenvironment (TME) in DLBCL affects patient prognosis (Nicholas et al., 2016; Ciavarella et al., 2018). Tumour-infiltrating lymphocytes (TILs) were first reported in 1986 by Rosenberg et al. (1986), and they are an important player in the immunomodulatory function of the TME. CD8^+^ T cells are the principal constituents of TILs, and CD8^+^ T cell dysfunction is the primary cause of tumour immune tolerance and escape (Jiang et al., 2021). Studies of the immune microenvironment of solid tumours suggested that tumours can be broadly classified into T cell-inflamed and non-T cell-inflamed phenotypes (Sharma and Allison, 2015; Gajewski et al., 2017; Thorsson et al., 2019). T cell-inflamed tumours are characterised by upregulation of the expression of T cell activation-associated genes and downstream genes of the interferon-γ signalling pathway (Tumeh et al., 2014; Ayers et al., 2017), greater T cell infiltration, and a stronger response to immune checkpoint blocking therapy (ICBT) (Zhang et al., 2020). In contrast, non-T cell-inflamed tumours are largely devoid of infiltrating immune cells and usually respond poorly to ICBT (Tang et al., 2020).

Many lymphoma subtypes have been found to be sensitive to ICBT, and all share increased T-cell infiltration as a common feature. Classic Hodgkin’s lymphoma (cHL) is a typical inflamed lymphoma with upregulated programmed cell death protein 1 (PD-1) expression in the TME, which binds to programmed cell death ligand 1 (PD-L1) on tumour cells to inhibit the effector function of CD8^+^ T cells (Liu and Shipp, 2017). Studies on PD-1 monoclonal antibody treatment for refractory or relapsed (r/r) cHL have shown that the effector functions of dysfunctional T cells are restored when the dominant immune checkpoints are blocked (Armand et al., 2015; Younes et al., 2016; Chen et al., 2017).

Unlike cHL, DLBCL is usually considered a non-inflamed lymphoma (Kline et al., 2020). First, germinal centre B-cell derived high grade B cell lymphoma is associated with MYC, BCL-2, and/or BCL6 rearrangements and the expression of double-hit genes, exhibiting sustained tumour cell proliferation, suppressing immune cell infiltration, and promoting immune “exclusion” (Ennishi et al., 2019). Second, DLBCL is enriched in EZH2-activating mutations, leading to the downregulation of HLA expression and promoting immunological “ignorance” (Ennishi et al., 2019). Similar phenomena have been observed in other cancers (Burr et al., 2019; Ennishi, Takata, et al., 2019). Therefore, ICBT is usually ineffective against DLBCL, though some studies have reported its successful use in r/rDLBCL (Xu-Monette et al., 2018; Zhang et al., 2020; Wang et al., 2021; Mu et al., 2022). It is thus speculated that an inflammatory environment favourable to ICBT might exist in some cases of DLBCL. Therefore, distinguishing between inflamed and non-inflamed phenotypes and understanding the mechanisms underlying the development of non-inflamed lymphoma will facilitate the design of treatment strategies that target phenotype switching to improve the efficacy of ICBT.

In this study, we analysed the immunological and prognostic characteristics of the DLBCL TME using ESTIMATE, CIBERSORT, and single-sample gene set enrichment analysis (ssGSEA) and investigated the regulatory mechanisms of the immune microenvironment. We aimed to find the hub genes associated with CD8^+^ T cells using weighted gene co-expression network analysis (WGCNA) and constructed immune-related gene scores (IRGSs) to assess the prognostic value and predict the efficacy of ICBT. The results showed that the IRGS is a good prognostic indicator and a useful tool for differentiating inflammatory phenotypes and predicting the efficacy of ICBT.

## Materials and methods

### Data collection

Gene expression data and corresponding clinical information were obtained from the Gene Expression Omnibus (GEO; https://www.ncbi.nlm.nih.gov/geo/) database. The datasets were GSE10846 (DLBCL = 414), GSE32918 (DLBCL = 172), GSE56315 (DLBCL = 55, normal = 33), and GSE12195 (DLBCL = 73, normal = 20). GSE10846 and GSE32918 were used as independent training and validation sets, where clinical feature parameters were obtained from GSE10846. Gene expression data from GSE56315 and GSE12195 were obtained from patients with DLBCL and normal subjects for differential gene analysis. Expression values were normalised by the data submitter. Immune-associated gene sets were downloaded from the ImmPort (https://www.immport.org/Shared/home) database.

### Identification of DLBCL subtypes based on immune gene sets

A literature search was performed to identify 29 immune-associated gene sets representing tumour immunity (He et al., 2018). ssGSEA was performed on the 29 immune gene sets using the R package “GSVA” (Hänzelmann et al., 2013) to obtain the ssGSEA score for each sample. The R package “ConensusClusterPlus” (Wilkerson and Hayes, 2010) was used for consistent clustering based on ssGSEA scores and immunosubtype screening (50 iterations with an 80% resampling rate). The optimal clustering number was determined by the k value of the cumulative distribution function (CDF) curve with the minimum descending slope. Principal component analysis (PCA) was performed using the R package “PCA” to verify the reliability of consistent clustering.

### Evaluation of immune cell infiltration, tumour purity, and matrix content in DLBCL

ESTIMATE is an algorithm used to determine the ratio of stromal cells to immune cells based on gene expression characteristics in tumour samples. The stromal score (stromal content), immune score (degree of immune cell infiltration), ESTIMATE score (combined stromal and immune scores), and tumour purity data were obtained for each sample using the R package “ESTIMATE” to compare the differences between immune subtypes.

### Heatmap

The ssGSEA score, ESTIMATE algorithm results, and immune subtypes of each sample were combined into a heatmap to visualise the TME differences among different immune subtypes. Heatmap visualisation was performed using the R package “heatmap”.

### Comparison of immune cell subgroups and GSEA

CIBERSORT (Newman et al., 2015) is a linear support vector regression-based deconvolution analysis method for unknown mixed cell populations and expression matrices containing similar cell types, which can be used to assess immune cell contents (Gentles et al., 2015). The infiltration levels of 22 types of immune cells in DLBCL were assessed using the R package “CIBERSORT” to compare differences between different immune subtypes. GSEA was performed on immune subtypes using GSEA version 4.0 with c2.cp.kegg.v7.5.1.symbols.gmt as the reference gene set. A normalised enrichment score (|NES| > 1) and FDR < 0.05 indicated significant pathway enrichment.

### Determination of CD8^+^ T cell-related hub genes

The differentially expressed genes (DEGs) between DLBCL and normal lymphocytes in the GSE56315 and GSE12195 datasets were analysed using the R package “limma” with the threshold values set to |log_2_ fold-change | > 2 and 1, respectively, and the adjusted *p*-value to < 0.05. WGCNA was performed on DEGs from both datasets using the R package “WCGNA” (Langfelder and Horvath, 2008). To obtain CD8^+^ T cell-related hub genes in DLBCL, we used the T cell content obtained from the CIBERSORT algorithm as phenotypic data. Soft threshold values of 6 and 5 were set for the GSE56315 and GSE12195 datasets, respectively, with a hub gene correlation threshold of 0.7, a threshold for module merging of 0.5, and a minimum module size of 20. In the end, the obtained modules were correlated with the phenotypic data to obtain the key modules associated with CD8^+^ T cells.

The two key module genes obtained from WCGNA were intersected with 1,793 immune genes from the IMMPORT (https://www.immport.org/) database to obtain immune genes associated with CD8^+^ T cells. These were imported into STRING (https://cn.string-db.org/) to construct a protein–protein interaction (PPI) network with the PPI threshold set to 0.7 to obtain hub genes highly associated with CD8^+^ T cells. Finally, Gene Ontology (GO) and Kyoto Encyclopaedia of Genes and Genomes (KEGG) enrichment analyses of the hub genes were performed using the R package “ClusterProfiler” (Yu et al., 2012), with FDR < 0.05 as the screening threshold.

### Dimension reduction and generation of IRGSs

SPSS 25.0 software was used to obtain standardised processed hub gene expression data *via* the Z-score method. The suitability of factor analysis was tested using the Kaiser–Meyer–Olkin (KMO) test and Bartlett’s sphericity test (IBM Knowledge Center, 2012). If the KMO value was close to 1 and Bartlett’s sphericity test had a *p-*value < 0.05, then the results of factor analysis were considered reliable. Dimensional reduction was performed on the hub genes, and the composite score of each sample was obtained based on the principal component coefficients and the principal component variance contribution of each gene; this was defined as the IRGS of each sample. The formulae are as follows:

$$Y=\overset{n}{\underset{i=1}{\sum }}\left(Xi\times ui\right)$$

$$Z=\overset{p}{\underset{j=1}{\sum }}\left(Yj\times bj\right)$$

where n is the number of hub genes, u_i_ is the principal component coefficient of gene i, X_i_ is the standardised data of gene i, p is the number of principal components, b_j_ is the variance contribution of principal component j, Y_j_ is the score of principal component j, and Z is the composite score.

### Immunotherapeutic response prediction

The training and validation set gene expression data were imported into the Tumour Immune Dysfunction and Exclusion (TIDE) web tool (http://tide.dfci.harvard.edu/) using the TIDE algorithm (Jiang et al., 2018) and subclass mapping (Hoshida et al., 2007) to compare gene expression data from subgroups differentiated based on their IRGS, with a dataset of 47 melanoma patients responding to immune checkpoint inhibitors (CTLA-4 and PD-1) (Roh et al., 2017), and to predict the response of different subgroups of patients to ICBT.

We retrospectively analysed 30 patients with r/rDLBCL who received second-line therapy combined with PD-1 inhibitors (camrelizumab and tislelizumab) between 2020 and 2022. Age, sex, stage, cell of origin, absolute lymphocyte count (ALC), and treatment protocol were collected. Treatment response was evaluated according to the Lugano 2014 assessment criteria (Cheson et al., 2014). The retrospective study was approved by the Ethics Committee of Guangxi Medical University Cancer Hospital.

### Verification of the prognostic value of hub genes

To verify the prognostic value of the hub genes, univariate Cox regression analysis was performed on the hub genes, and genes significantly associated with prognosis (*p* < 0.05) were screened. Least absolute shrinkage and selection operator (LASSO) regression analysis was performed using the R package “glmnet” to screen for genes highly associated with prognosis based on the best lambda value to establish a risk model, and the risk score for each patient was calculated using the coefficients obtained from the LASSO algorithm, as follows:

$$Risk\;score=\overset{n}{\underset{i=1}{\sum }}\left(Expi\times Coefi\right)$$

where n is the number of prognostic genes, Exp_i_ is the expression of gene i, and Coef_i_ is the regression coefficient of gene i in the LASSO algorithm (Tibshirani, 1997). The patients were divided into high- and low-risk groups using the best cut-off value, and the difference in survival periods between the two groups was assessed using the R package “survival”. Finally, the R package “survivalROC” was used to calculate the area under the curve (AUC) of receiver operator characteristic (ROC) curves to assess the accuracy of the prognostic determination (Blanche et al., 2013).

### Statistical analysis

All statistical analyses were performed using R software (v3.5.2). Kaplan–Meier curves and log-rank tests were used to compare the overall survival (OS) of patients with DLBCL between different groups. For data conforming to a normal distribution, a *t*-test was used for comparisons of two independent samples, and the Pearson test was used for correlation analysis. Otherwise, the Wilcoxon test and Spearman test were used. All statistical tests were two-sided, and differences with *p* < 0.05 were considered statistically significant.

## Results

### Identification of DLBCL subtypes based on immune gene sets

**FIGURE 1:**
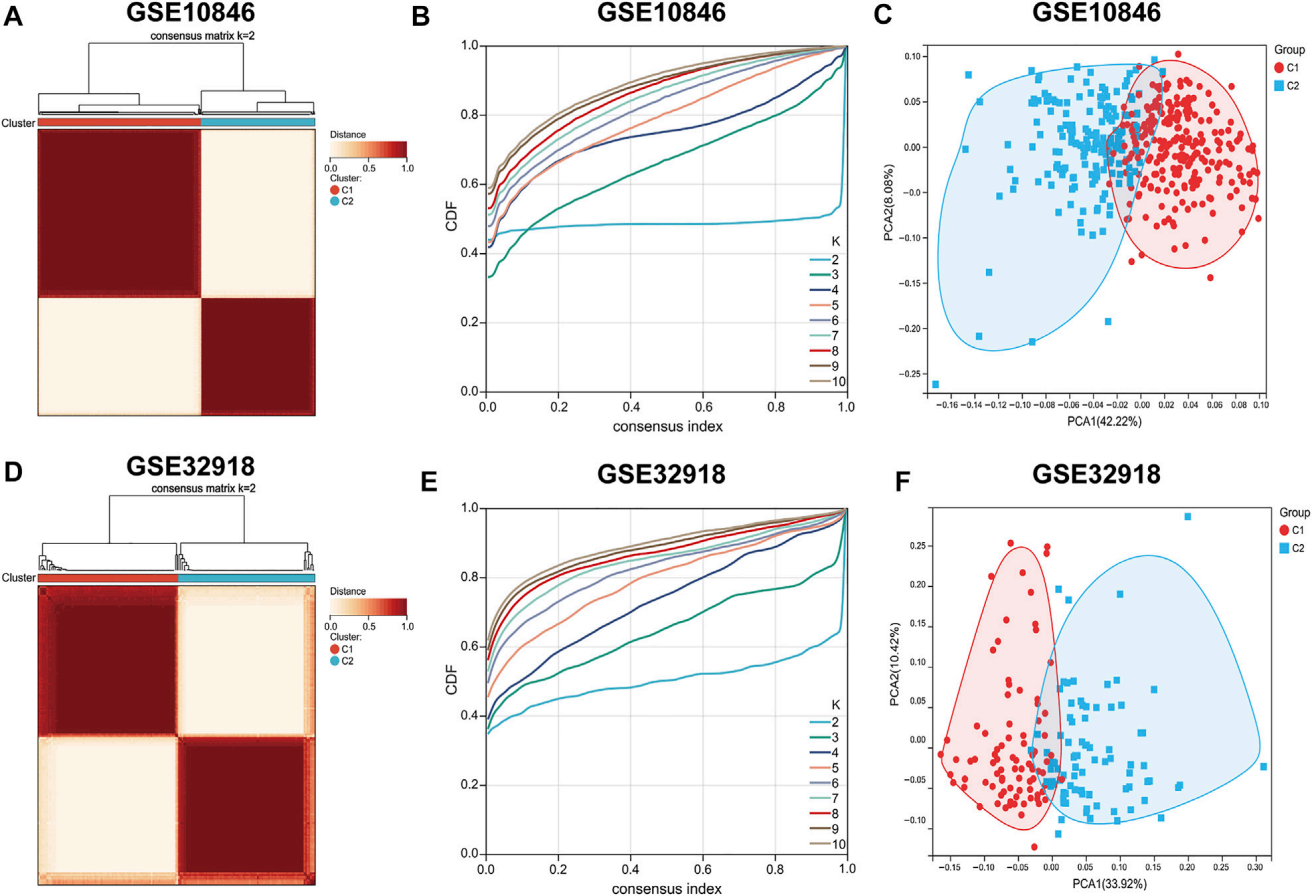
Consensus clustering of DLBCL patients. **(A–D)** Consensus matrix heatmaps indicating that the optimal value for consensus clustering is *K* = 2 both in the training and validation sets. **(B,E)** Cumulative distribution function (CDF) (*K* = 2–10). **(C–F)** Principal component analysis (PCA) based on ssGSEA scores of the training and validation sets. Each point represents a sample, and different colors distinguish immune subtypes.

To score the 29 immune gene sets for each sample in the training set, ssGSEA was used (Supplementary Table S1). The R package “ConsensusClusterPlus” was used to classify all samples into k subtypes (*k* = 2–10). Based on the CDF curve and clustering heat map, *k* = 2 is optimal (i.e., all samples can be divided into two clusters). To verify the reliability of consistent clustering, PCA showed that patients with DLBCL could be classified into two subtypes based on the ssGSEA score (designated C1 and C2, Figures 1A–C, Supplementary Table S2); the same results were obtained with the validation set (Figures 1D–F, Supplementary Tables S3, S4).

### Immune characteristics of the two immune subtypes

**FIGURE 2:**
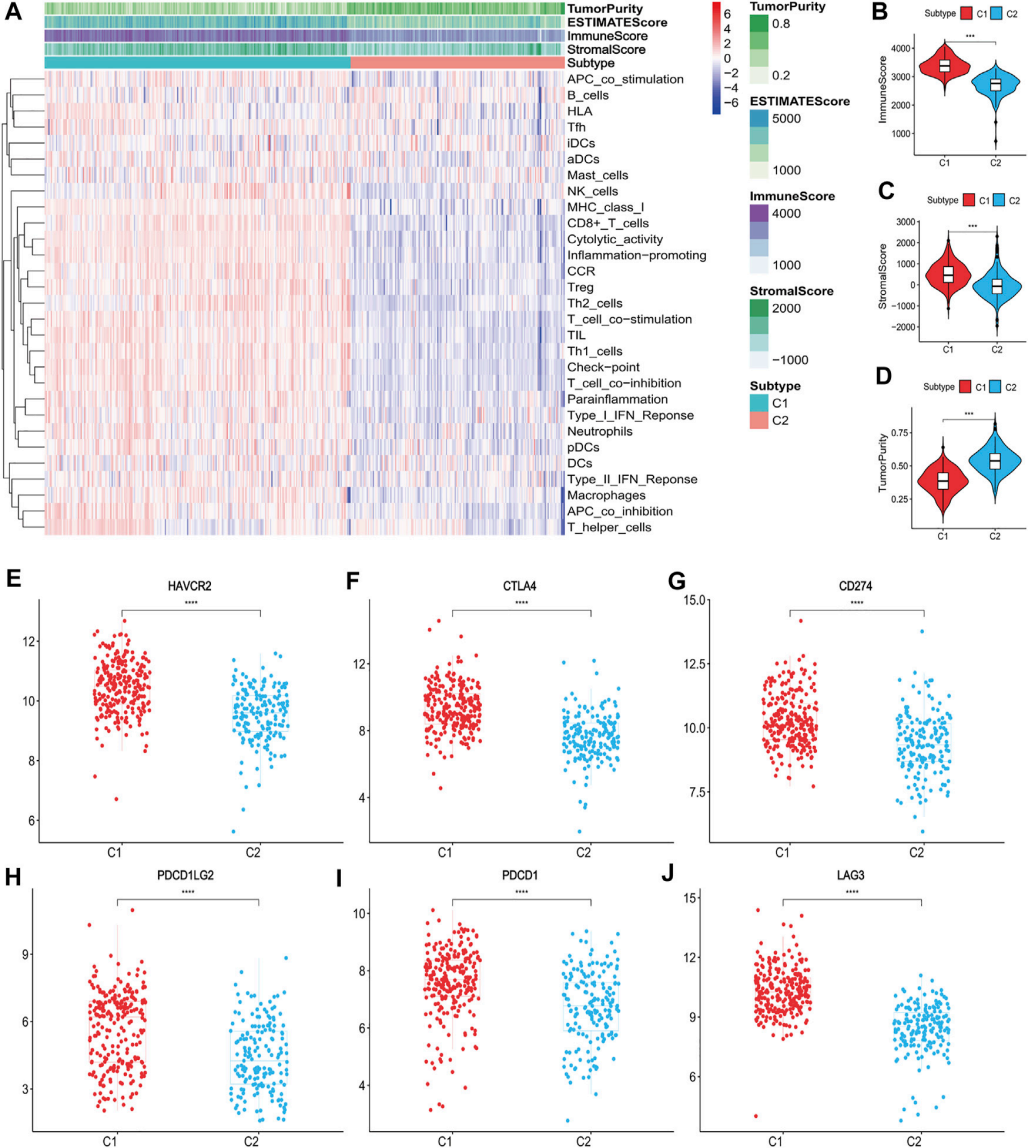
Identification of the two subtypes in the training set. **(A)** Heatmap of the two subtypes based on ssGSEA scores for 29 immune gene sets. **(B–D)** Evaluation of stromal scores, immune scores, ESTIMATE scores and tumor purity for the two subtypes. **(E–J)** The expression differences of immune checkpoint genes between the two subtypes; Bars indicate medians. Wilcoxon test was used to compare gene expression levels between the two subtypes. ***p < 0.001, ****p < 0.0001.

**FIGURE 3:**
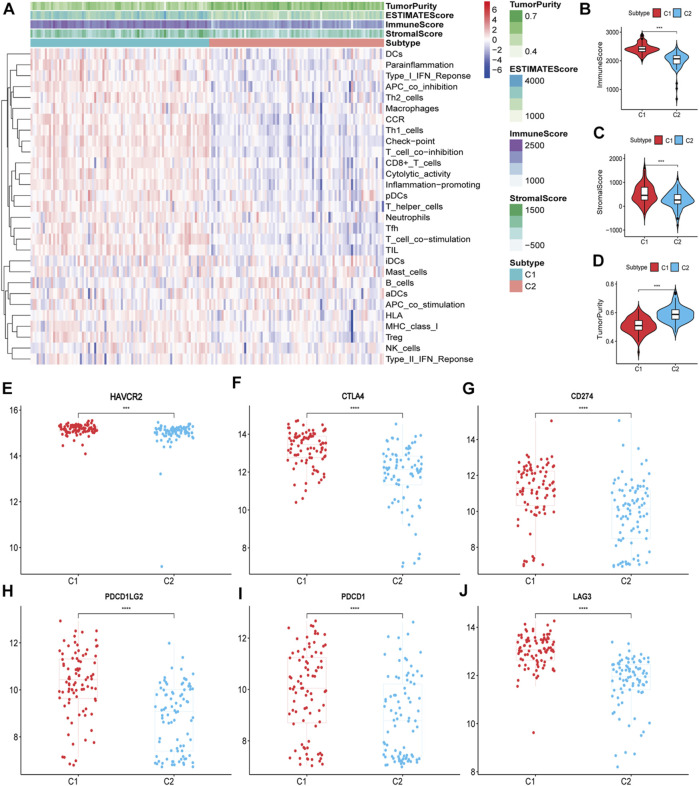
Identification of the two subtypes in the validation set. **(A)** Heatmap of the two subtypes based on ssGSEA scores for 29 immune gene sets. **(B–D)** Evaluation of stromal scores, immune scores, ESTIMATE scores and tumor purity for the two subtypes. **(E–J)** The expression differences of immune checkpoint genes between the two subtypes; Bars indicate medians. Wilcoxon test was used to compare gene expression levels between the two subtypes. ***p < 0.001, ****p < 0.0001.

The ssGSEA score heatmaps for the 29 immune gene sets showed that the degree of immune infiltration was greater in subtype C1 than in subtype C2 (Figures 2A, 3A). Here, the ssGSEA scores for CD8^+^ T cells, immune checkpoints, the class I major histocompatibility complex (MHC-I), cytolytic activity, macrophages, Th1 cells, and other immune components were significantly higher in subtype C1, whereas the scores for B cells were higher in subtype C2 (Supplementary Figures S1, S2). ESTIMATE results showed higher stromal scores, immune scores, and ESTIMATE scores in subtype C1 and conversely, higher tumour purity in subtype C2 (Figures 2B–D, 3B–D, Supplementary Tables S5, S6). Finally, we examined the expression of six immune checkpoint genes associated with immune escape (*PDCD1*, *CD274*, *PDCD1LG2*, *CTLA4*, *HAVCR2*, and *LAG3*), and the results showed higher expression levels in subtype C1 (Figures 2E–J, 3E–J). In summary, the content of immune cells in subtype C1 was greater than that in subtype C2, and conversely, the content of tumour cells in subtype C2 was greater than that in subtype C1.

### Comparison of 22 immune cell types between the two subtypes and GSEA

**FIGURE 4:**
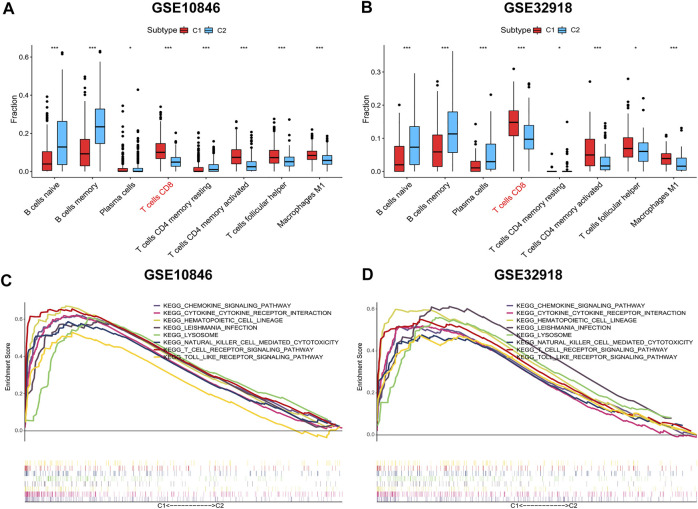
Distribution of immune cells and gene sets enrichment analysis (GSEA). **(A-B)** The distribution differences of immune cells between the subtypes based on CIBERSORT algorithm. **(C-D)** C1 vs. C2 GSEA. Wilcoxon test was used to compare immune cells of the two subtypes. *p < 0.05, **p < 0.01, ***p < 0.001.

To compare the differences in the distribution of immune cells between immune subtypes, the CIBERSORT algorithm was used to calculate the contents of 22 immune cell types (Supplementary Figures S3, S4, Supplementary Tables S7, S8). In the training and validation sets, CD8^+^ T cells, CD4^+^ resting memory T cells, CD4^+^ activated memory T cells, and follicular helper T cells were more prevalent in subtype C1, whereas naive B cells, memory B cells, and plasma cells were more abundant in subtype C2 (Figures 4A,B). This was consistent with ssGSEA and ESTIMATE results.

Next, the enriched pathways associated with the immune subtypes were analysed to explore the biological function of immune subtypes. Using an FDR <0.05 as the screening criterion, the top 10 GSEA-enriched pathways in the training and validation sets were identified, of which eight enriched pathways were shared between the two subtypes (lysosome, T cell receptor, chemokine, cytokine_cytokine, natural killer cell-mediated cytotoxicity, hematopoietic cell lineage, *Leishmania* infection, and Toll-like receptor (TLR) signal pathways; Figures 4C,D, Supplementary Tables S9, S10). The co-enriched pathways in subtype C2 were DNA replication and mismatch repair pathways, but the enrichment was not significant (FDR >0.05).

### Prognostic significance of the two immune subtypes

**FIGURE 5:**
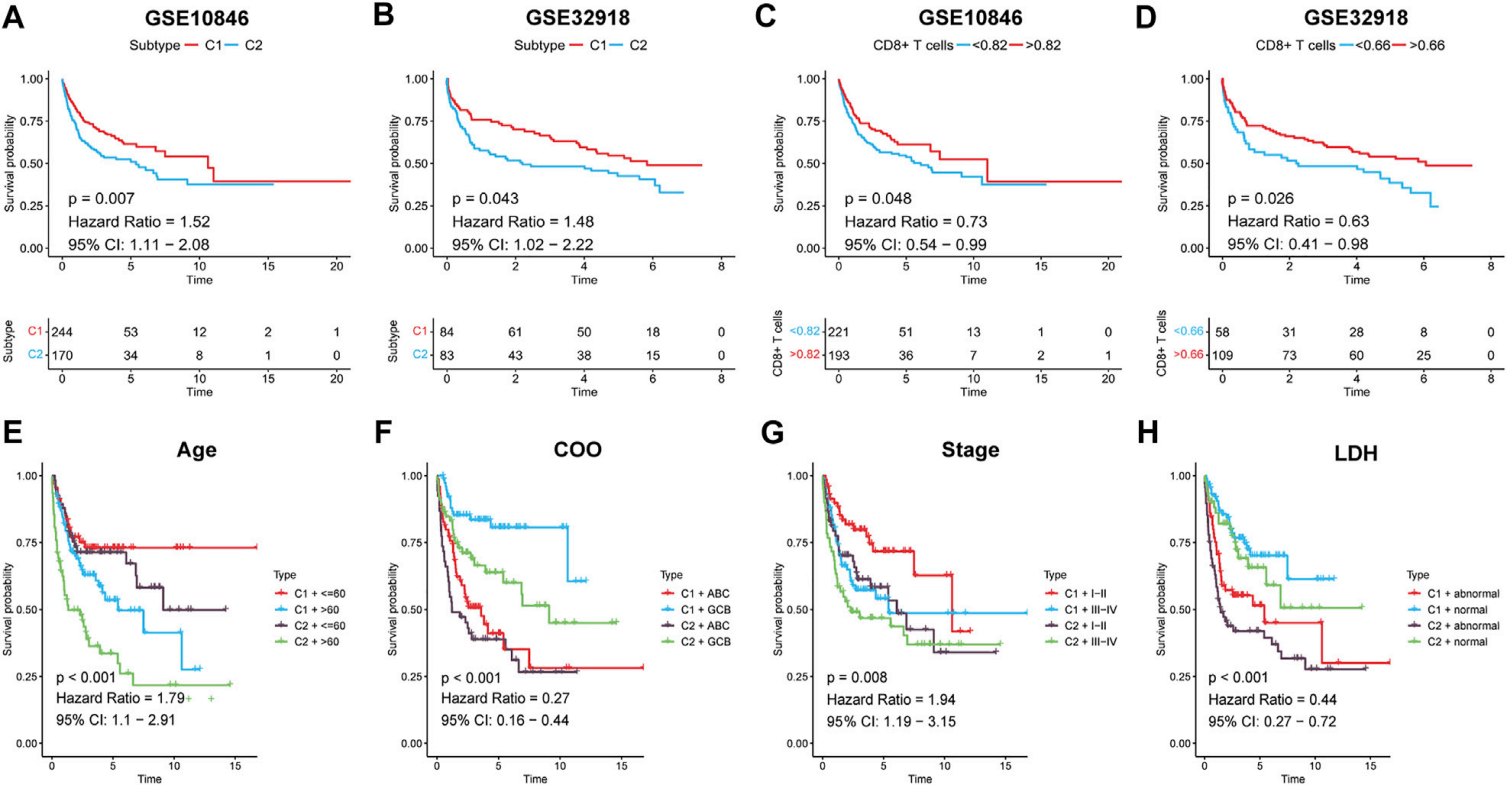
Survival analysis for DLBCL patients. **(A,B)** Kaplan-Meier survival curves for the two subtypes. **(C,D)** Kaplan-Meier survival curves for high/low CD8^+^ T cells groups. Kaplan-Meier survival curves for DLBCL patients both by the two subtypes and **(E)** Age, **(F)** COO, **(G)** Stage, **(H)** LDH, respectively. *P* < 0.05 was considered significant.

To assess the effect of immune infiltration on prognosis, we analysed the relationship between immune subtypes and prognosis based on the clustering results of immune gene sets. In the training set, Kaplan–Meier curve analysis showed that better OS was associated with subtype C1 (HR: 1.52, 95% CI: 1.11–2.08, *p* = 0.007; Figure 5A). CD8^+^ T cells are the principal component of the TME and participate in anti-tumour immunity through cytotoxic T cell actions, and thus, increased CD8^+^ T cells were determined to be a favourable prognostic factor (HR: 0.73, 95% CI: 0.54–0.99, *p* = 0.048; Figure 5C). The same results were obtained in the validation set (Figures 5B,D). Age, cell-of-origin, stage, and lactic dehydrogenase have been consistently associated with DLBCL prognosis, and the combination of immunophenotyping and an analysis of these four clinical parameters allowed for more accurate prognostic stratification (Figures 5E–H, Supplementary Table S11). In conclusion, high immune cell infiltration favours patient survival.

### Gene mutations contribute to loss of MHC-I expression in DLBCL

The presentation of bound antigenic peptides by MHC-I to CD8^+^ T cells is a key step in the cytotoxic effects of CD8^+^ T cells. In addition, the antitumour effects of ICBT are dependent on CD8^+^ T cells, and specifically, the MHC-I-dependent immune response. Therefore, tumour cells with the deletion or downregulated expression of MHC-I evade T-cell recognition, which is one of the principal causes of ICBT resistance (Gu et al., 2021). Among B-cell lymphomas, MHC-I deletion is most common in DLBCL (46.2%) (Fangazio et al., 2021), which could be the primary reason for the associated poor efficacy of immunotherapy for DLBCL.

**FIGURE 6:**
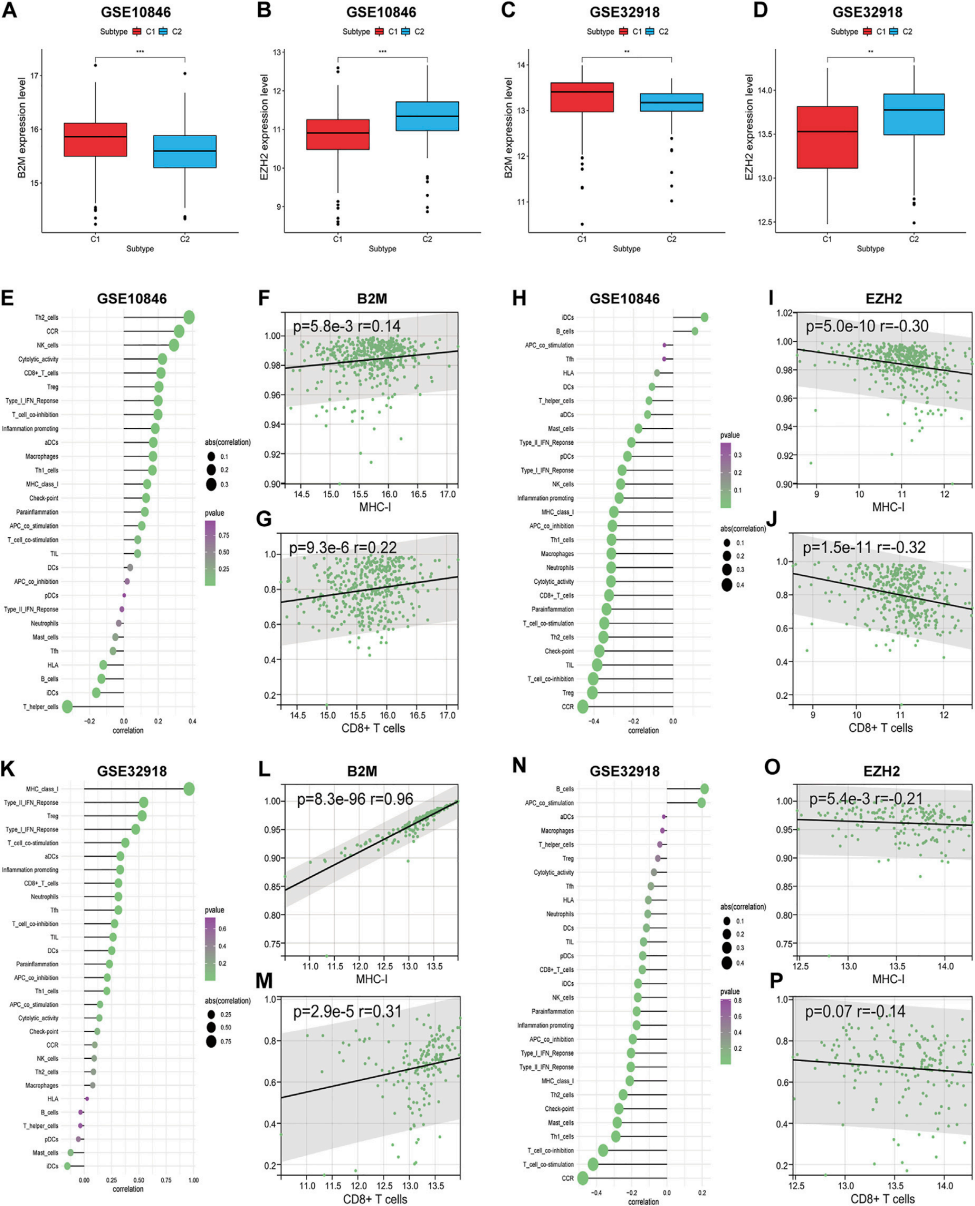
The correlations between the expression levels of two genes (*B2M* and *EZH2*) and DLBCL immune microenvironment. **(A–D)** The expression differences of two genes between the two subtypes. **(E,K)** The correlations between *B2M* and immune cell contents. **(H,N)** The correlations between *EZH2* and immune cell contents. **(F,G) (L,M)** The correlations between *B2M* and MHC-I, CD8^+^ T cells, respectively. **(I,J) (O,P)** The correlations between *EZH2* and MHC-I, CD8^+^ T cells, respectively. Wilcoxon test was used to compare gene expression levels between the two subtypes. Spearman test for correlational analyses. **p < 0.01, ***p < 0.001.

B2M is known as an important subunit of the MHC molecule, and mutations in B2M lead to reduced MHC synthesis (Fangazio et al., 2021). EZH2 (a histone methyltransferase) has been shown to promote immune escape by inhibiting MHC-I-mediated antigen presentation in a variety of tumours (Hogg et al., 2020). In addition, mutations in *B2M* and *EZH2* are common in DLBCL (Catalogue Of Somatic Mutations In Cancer, 2020). Upon investigating the regulatory mechanism of MHC-I in DLBCL, we found that B2M expression in the training set was significantly higher in subtype C1 than in subtype C2 (*p* < 0.001), and EZH2 expression was even more highly expressed in subtype C2 (*p* < 0.001; Figures 6A,B). Further correlation analysis showed that B2M and EZH2 expression levels were significantly positively (*r* = 0.14, *p* < 0.01) and negatively (*r* = −0.30, *p* < 0.001) correlated with MHC-I, respectively (Figures 6F,I). We also found that gene expression levels correlated with the degree of immune infiltration (Figures 6E,H). Specifically, B2M and EZH2 expression levels were significantly positively (*r* = 0.22, *p* < 0.001) and negatively (*r* = −0.32, *p* < 0.001) correlated with CD8^+^ T cells, respectively (Figures 6G,J). The same results were obtained in the validation set (Figures 6C,D,K–P). Thus, we speculate that *B2M* and *EZH2* mutations are the primary cause of MHC-I downregulation in DLBCL and are also involved in the regulation of immune infiltration.

### Determination of CD8^+^ T cell-related hub genes

**FIGURE 7:**
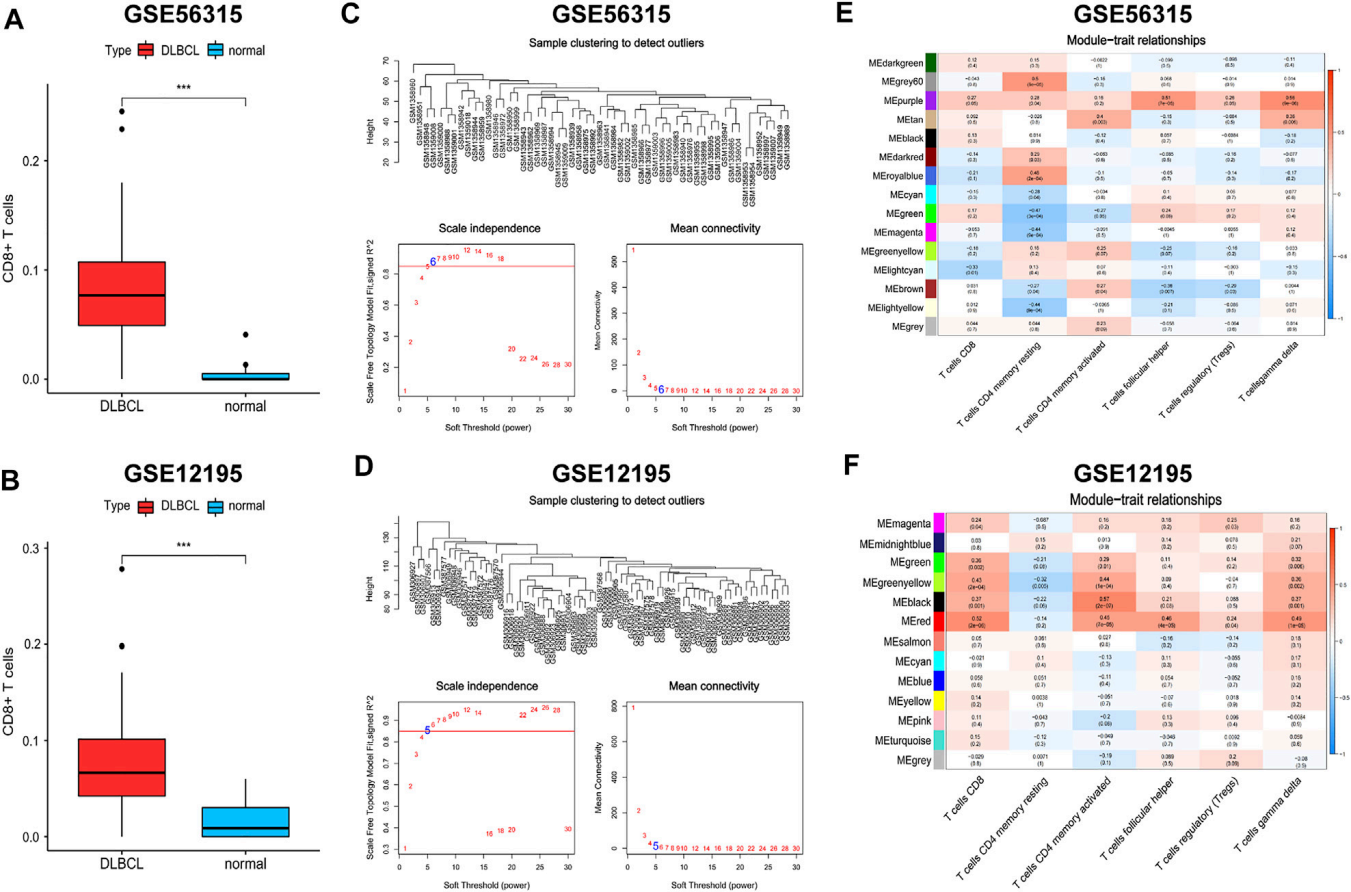
Weighted gene coexpression network analysis (WGCNA). **(A,B)** The distribution differences of CD8^+^ T cells between DLBCL and normal patients. **(C,D)** Topological network analysis of the optimal soft threshold. **(E,F)** Identification of weighted gene co-expression network modules associated with CD8^+^ T cells. Wilcoxon test for comparison of CD8^+^ T cells distribution between DLBCL and normal patients. ***p < 0.001.

CD8^+^ T cells are the main component of TILs in the TME and are closely related to anti-tumour immunity and immune escape (Jiang et al., 2021). To obtain CD8^+^ T cell-related hub genes, we first assessed the level of immune infiltration in patients with DLBCL and normal subjects based on the GSE56315 and GSE12195 datasets using the CIBERSORT algorithm (Supplementary Figures S5, S6). CD8^+^ T cell levels were significantly elevated in patients with DLBCL (Figures 7A,B). We then performed WCGNA on the DEGs obtained using the “limma” package (Supplementary Tables S12, S13). Because the proportion of CD4^+^ naive T cells was nearly zero in each sample, we extracted data on the proportions of CD8^+^ T cells, CD4^+^ resting memory T cells, CD4^+^ activated memory T cells, follicular helper T cells, regulatory T cells, and γδ T cells as phenotypic data and performed association analysis with the modules obtained from WCGNA (Supplementary Tables S14, S15). The “purple” module genes of GSE56315 (152 genes, Supplementary Table S16) and the “red” module genes of GSE12195 (266 genes, Supplementary Table S17) had the highest correlation with CD8^+^ T cells and a consistent positive correlation (Figures 7C–F).

**FIGURE 8:**
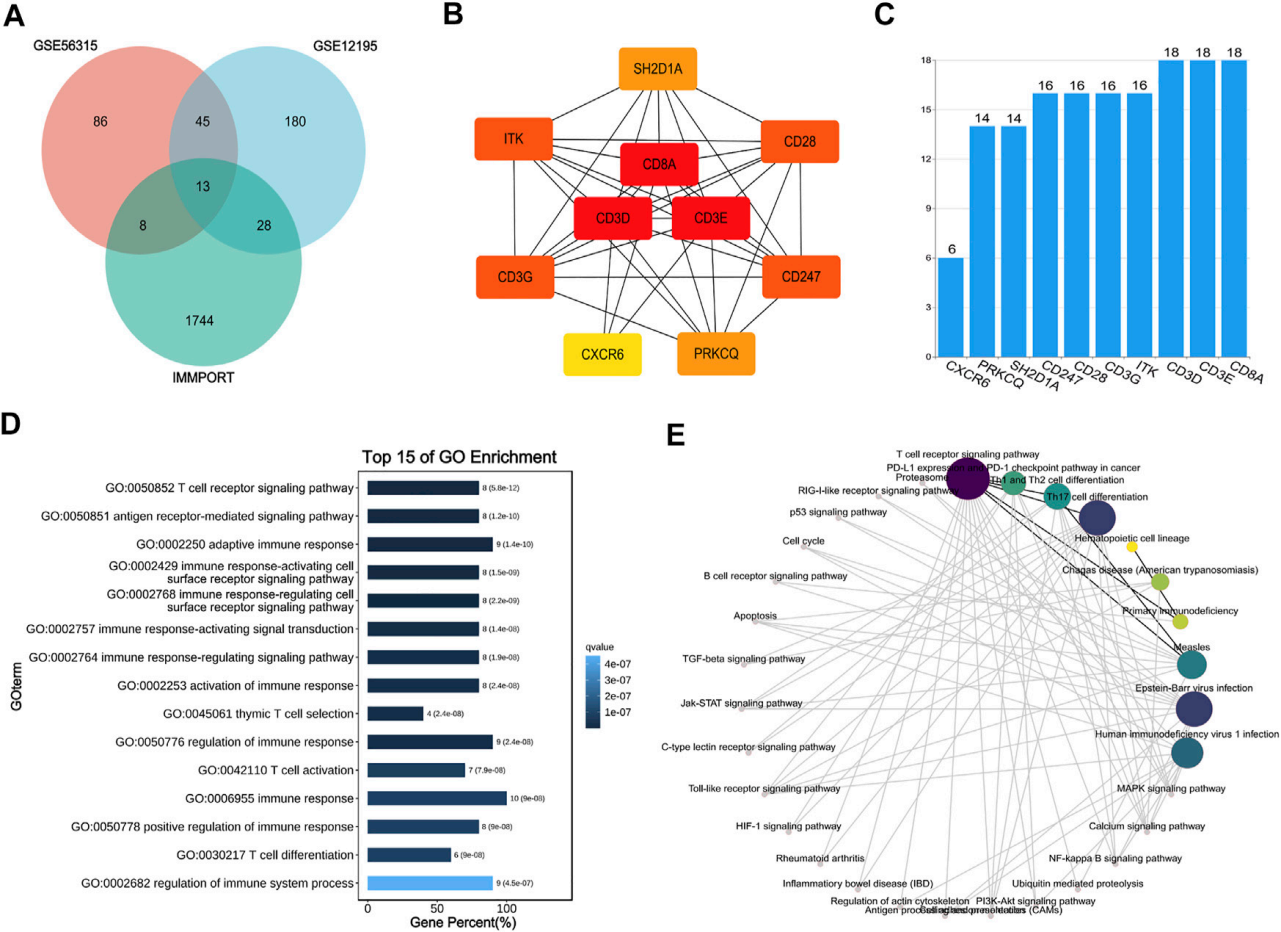
Interaction network and enrichment analysis of hub genes. **(A)** Venn diagram of GES56315, GSE12195 and IMMPORT database. **(B)** Protein-protein interaction (PPI) network of 10 hub genes. **(C)** The histogram for number of gene-gene interactions. **(D,E)** GO and KEGG enrichment analysis of 10 hub genes.

The genes in these two key modules were cross-referenced with 1,793 immune genes downloaded from the IMMPORT database to obtain 13 hub genes associated with CD8^+^ T cells (*SH2D1A*, *CD8A*, *GBP2*, *ITK*, *CD3D*, *RORA*, *IL1R2*, *CD28*, *CD247*, *CD3G*, *PRKCQ*, *CXCR6*, and *CD3E*). These 13 hub genes were imported into the STRING database to build a PPI network, and the interaction threshold was set to 0.7. Three genes (*RORA*, *GBP2*, and *IL1R2*) were removed, and in the end, a network of 10 hub genes was constructed with *CD3A*, *CD3D*, and *CD3E* as the core (Figures 8A–C).

To understand the biological processes and pathways associated with the effects of the hub genes in DLBCL, GO and KEGG enrichment analyses were performed. GO analysis showed that the hub genes were primarily involved in the immune response and T cell regulation. KEGG analysis showed that the hub genes act primarily on the T cell receptor, PD-1/PD-L1 immune checkpoint, and Th cell differentiation signalling pathways (Figures 8D,E).

### Correlation between the hub genes and immune microenvironment

**FIGURE 9:**
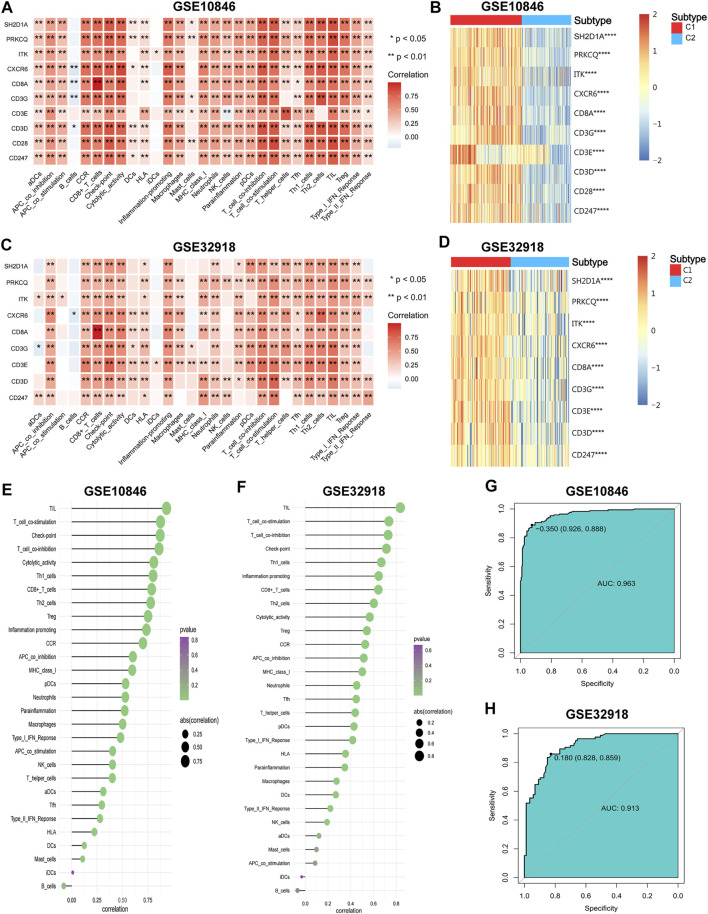
The correlations of hub genes and DLBCL immune microenvironment. **(A–C)** The correlations of hub genes and immune cell contents. **(B–D)** The expression differences of hub genes between the two subtypes. **(E,F)** The correlations of IRGSs and immune cell contents. **(G,H)** Receiver operator characteristic (ROC) curves for IRGS to predict immune subtypes. Wilcoxon test for comparison of gene expression levels between the two subtypes. Spearman test for correlational analyses. *p < 0.05, **p < 0.01, ***p < 0.001, ****p < 0.0001.

To further confirm the correlation between the hub genes and the immune microenvironment, correlation analysis between the training and validation sets was performed. The results showed that the hub genes were significantly and positively correlated with the ssGSEA scores of most immune gene sets, and the highest correlation was found with T cells (Figures 9A,C). The expression of all hub genes was significantly elevated in subtype C1 (Figures 9B,D).

To obtain the IRGS for each sample, dimensional reduction of the hub genes was performed. First, suitability tests showed good reliability of the results for factor analysis (KMO = 0.942 for the training set, KMO = 0.823 for the validation set, Bartlett’s sphericity test *p* < 0.001 for both). Then, factor analysis was performed to obtain the IRGS for each sample to represent the degree of immune infiltration (Supplementary Tables S18, S19). IRGSs showed a significant positive correlation with most immune components and the highest correlation with TILs (Figures 9E,F). Finally, ROC curve analysis showed that IRGSs could be used to accurately distinguish between different immune subtypes (AUC = 0.963 for the training set, 0.913 for the validation set; Figures 9G,H). In summary, the hub genes might positively regulate the inflammatory microenvironment, and the IRGS can reflect the degree of immune infiltration well and is a simple and useful tool to differentiate between immune subtypes.

### Immunotherapeutic response prediction

**FIGURE 10:**
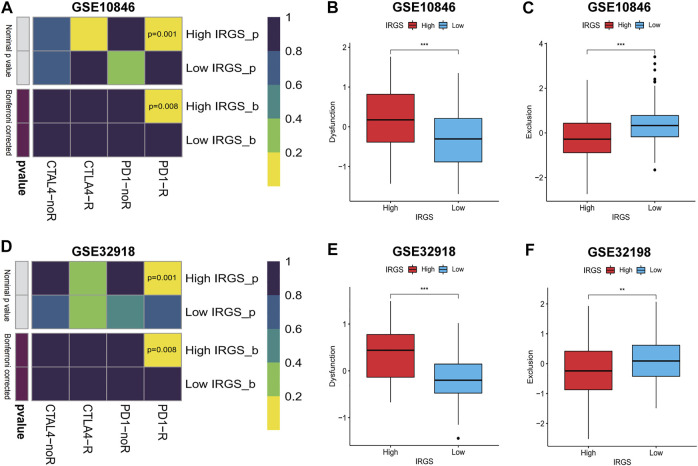
Predicting response to immune checkpoint blockade therapy (ICBT) and characteristics of T cells. **(A–D)** IRGS for predicting response to ICBT between high/low IRGS groups. **(B,C) (E,F)** The characteristic of T cells in high/low IRGS groups. Wilcoxon test for comparison of two independent samples. Spearman test for correlational analyses. **p < 0.01, ***p < 0.001.

ICBT has been approved as a routine treatment for a variety of tumours. Although some patients with DLBCL exhibit a response to ICBT, most studies have shown discouraging results (Hatic et al., 2021). Therefore, accurate predictors of the ICBT response would be beneficial for dosage guidance. We classified the samples into high- and low-IRGS groups based on the median IRGS and used the TIDE algorithm and subclass mapping to compare the expression profiles of the DLBCL subgroups with a dataset containing 47 melanoma patients responding to immune checkpoint inhibitors published by Roh et al. (2017). The high-IRGS group was more sensitive to ICBT and PD-1 monoclonal antibodies, which was statistically significant (Bonferroni correction *p* = 0.008). Further analysis of T cell functions and infiltration levels in the high/low-IRGS groups showed a higher “dysfunction” score in the high-IRGS group, suggesting that T cells were generally dysfunctional. However, the “exclusion” score was higher in the low-IRGS group, suggesting that T-cell rejection led to reduced infiltration (Figure 10).

**FIGURE 11:**
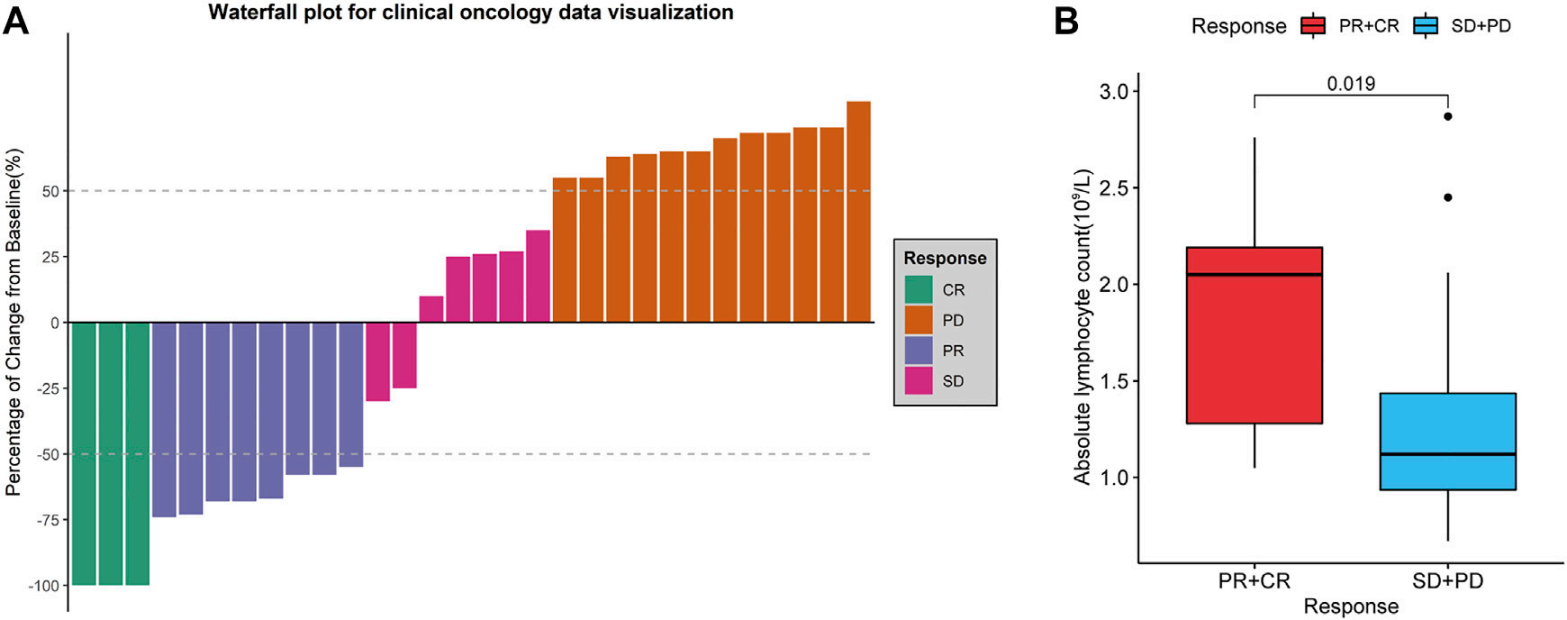
Treatment evaluation of 30 r/rDLBCL patients. **(A)** Waterfall plot for treatment evaluation. **(B)** Absolute lymphocyte count (ALC) in different treatment response groups. Wilcoxon test for comparison of ALC between the two groups.

We retrospectively analysed 30 patients with r/rDLBCL. The clinical data and treatment responses are shown in Table 1. The overall response rate was 36.7%, and the complete response rate was 10%. Patients who responded to treatment (complete response and partial response) had a significantly higher ALC than those who did not respond to treatment (stable disease and progressive disease) (*p* = 0.019) (Figure 11).

**TABLE 1:** Clinical characteristics of 30 r/rDLBCL patients with second-line treatments with PD-1 inhibitor.

ID	Age	Sex	Stage	COO	ALC(10^9^/L)	Treatment	Response
1	55	F	IV	GC	0.78	R-DHAP+PD-1	PD
2	60	M	III	NGC	1.75	R-GemoX+PD-1	PD
3	65	F	II	NGC	1.33	R-GemoX+PD-1	PR
4	68	F	IV	GC	1.45	R-ICE+PD-1	PD
5	71	M	IV	GC	0.75	R+PD-1	PD
6	63	F	IV	NGC	0.67	R-GemoX+PD-1	PD
7	78	M	III	GC	1.42	R+PD-1	SD
8	62	M	II	GC	1.12	R-GemoX+PD-1	SD
9	48	M	IV	NGC	1.23	R-ICE+PD-1	PR
10	61	F	II	NGC	1.02	R-GemoX+PD-1	PD
11	62	M	II	NGC	1.26	R-DHAP+PD-1	PD
12	67	F	III	NGC	0.95	RB+PD-1	SD
13	33	F	III	GC	2.19	R-DHAP+PD-1	CR
14	76	M	II	NGC	1.12	R-GemoX+PD-1	PD
15	51	F	IV	NGC	1.12	R-DHAP+PD-1	SD
16	55	M	II	GC	2.76	RB+PD-1	PR
17	62	M	III	NGC	2.54	R-GemoX+PD-1	PR
18	34	M	II	GC	1.45	R-GemoX+PD-1	CR
19	30	F	IV	NGC	0.89	R-ICE+PD-1	PD
20	69	M	III	GC	2.87	R-ICE+PD-1	SD
21	72	M	III	NGC	1.16	R+PD-1	SD
22	62	M	II	GC	1.05	R-DHAP+PD-1	PR
23	65	M	III	NGC	2.19	R-GemoX+PD-1	PR
24	62	F	II	GC	2.06	R-GemoX+PD-1	PR
25	62	M	III	NGC	2.05	R-GemoX+PD-1	PR
26	68	F	III	NGC	1.13	R-GemoX+PD-1	PR
27	44	M	III	NGC	2.45	R-GemoX+PD-1	PD
28	66	F	II	GC	1.12	R-ICE+PD-1	PD
29	36	F	II	NGC	2.05	R-DHAP+PD-1	CR
30	68	F	IV	GC	0.92	R-GemoX+PD-1	SD

COO, cell of origin; F, female; M, male; ALC, absolute lymphocyte count; PD, progressive disease; PR, partial response; SD, stable disease; CR, complete response; GC, germinal centre; NGC, non-germinal centre; R, rituximab; B, bendamustine; DHAP, dexamethasone, High-dose cytarabine, cis-platinum; GemoX, gemcitabine, oxaliplatin; ICE, ifosfamide, carboplatin, etoposide; PD-1, programmed cell death protein 1.

### Verification of the prognostic value of hub genes

**FIGURE 12:**
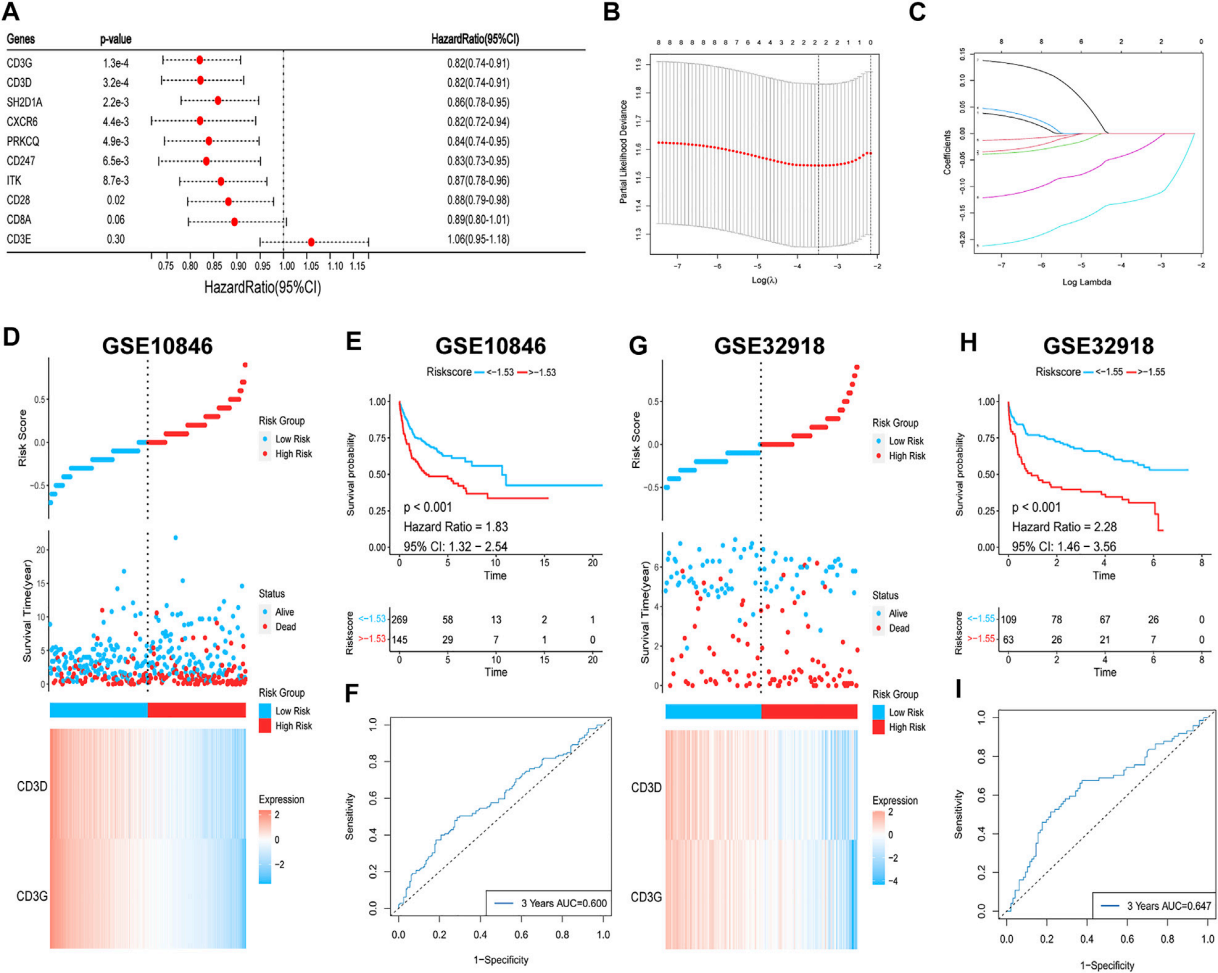
Survival analysis of hub genes. **(A)** Univariate Cox regression. **(B,C)** Lasso regression. **(D,E) (G,H)** Risk heatmaps and Kaplan-Meier survival curves of riskscores. **(F–I)** ROC curves for predicting 3-year OS based on riskscores.

To verify the relationship between the hub genes and prognosis, one-way Cox regression analysis of the 10 hub genes in the training set was performed, which showed that high expression of eight genes (*SH2D1A*, *ITK*, *CD3D*, *CD28*, *CD247*, *CD3G*, *PRKCQ*, and *CXCR6*) was significantly associated with elevated OS (Figure 12A). To avoid overfitting, LASSO regression was performed, which identified *CD3G* and *CD3D* as highly correlated with prognosis (Figures 12B,C). These two genes were used to construct risk models based on the minimum criteria, and risk scores were calculated based on LASSO regression coefficients using the following functions:

Risk score=−0.127×CD3G−0.033×CD3D

The patients were then classified into high- and low-risk groups based on the risk score. Survival analysis showed that OS was significantly lower in the high-risk group than in the low-risk group (*p* < 0.001; Figures 12D,E). The ROC curve was also plotted to assess the predictive effect of the risk score on prognosis, and the AUC for the 3-year OS was 0.600 (Figure 12F). The risk model yielded the same results with the validation set, with worse OS in the high-risk group (*p* < 0.001; Figures 12G,H) and an AUC for the 3-year OS of 0.647 (Figure 12I). In summary, a higher level of hub gene expression was indicative of better prognosis, and the risk model constructed based on the training set has moderate accuracy for prognostic assessment.

## Discussion

DLBCL is a highly aggressive and heterogeneous tumour, and although rituximab-based therapy has led to a significantly improved prognosis for patients, a breakthrough therapeutic approach is needed for recurrent or refractory patients. Tumours can be classified as ‘hot’ or ‘cold’ tumours based on the distribution of CD8^+^ T cells in TME. Solid tumour studies have shown that hot tumours respond well to immunotherapy, and ICBT has become a routine therapy for a variety of tumours (Mai et al., 2021; Paz-Ares et al., 2022; Qu et al., 2022). However, most studies of DLBCL with respect to ICBT have yielded disappointing results (Hatic et al., 2021), which could be explained by the fact that most cases of DLBCL comprise cold tumours. An analysis of GEO datasets by Wang et al. (2021) showed that DLBCL can also be differentiated into high- and low-immune infiltrative subtypes. However, this study was deficient in that 1) no external validation of the findings was performed, and 2) it failed to test the predictive value of immune subtypes in immunotherapy. In conclusion, studies on DLBCL immune subtypes are still lacking. Our study aimed to type DLBCL based on specific immune gene sets, analyse the immune and prognostic characteristics of different subtypes, investigate the regulatory mechanisms of immune subtypes, and assess the predictive value of IRGSs constructed based on CD8^+^ T cell-related hub genes for ICBT. In the end, the relationship between hub genes and prognosis was also analysed.

We classified DLBCL cases into subtypes C1 and C2 through consistent clustering, and immune profiling showed that subtype C1 is highly immune infiltrative, whereas the C2 subtype is poorly immune infiltrative. Subtypes C1 and C2 are associated with upregulation and downregulation of the expression of many immune-stimulating genes and the MHC, respectively. Aptsiauri et al. (2018) indicated that the shift from HLA-I-positive to HLA-I-negative in primary tumours is one of the primary mechanisms by which tumours evade recognition and destruction by T cells. The MHC-I complex consists of B2M and one of the HLA-I heavy-chain (hcHLA-I) molecules (Zinkernagel and Doherty, 1974a, 1974b; Townsend et al., 1985). The αβ receptors of cytotoxic CD8^+^ T cells recognise antigenic peptides presented by the MHC-I complex, leading to the destruction of target cells (Yewdell et al., 2003). Of note, 29% of DLBCL cases are accompanied by loss-of-function (LOF) mutations in B2M, one of the most common gene mutations (Chapuy et al., 2018; Schmitz et al., 2018). The present study showed that the expression levels of both B2M and MHC-I were downregulated in subtype C2, and the two were significantly positively correlated, and thus, we hypothesised that LOF mutations in B2M were responsible for the downregulation of MHC-I in subtype C2. Fangazio et al. (2021) identified 17 B2M double-allele (*n* = 11) and single-allele (*n* = 6) mutations and losses in genes in 42 MHC-I-negative DLBCL samples, accompanied by the disruption of one or more double alleles of the major hcHLA-I in four cases (9.4%). The present study demonstrates that both *B2M* and/or *HLA-I* gene inactivation can lead to MHC-I expression deficiency in DLBCL, consistent with our hypothesis.

MHC-I expression can be regulated by multiple factors. MHC-I-negative cases among patients with DLBCL are significantly more prevalent than negative cases owing to B2M mutations (75% vs. 43%, respectively), suggesting the existence of additional causes of the downregulation of MHC expression (Ennishi, Takata, et al., 2019). Ennishi, Takata, et al. (2019) found that gain-of-function (GOF) mutations in EZH2 are significantly increased in MHC-I- and MHC-II-negative patients with DLBCL. Through *in vivo*/*ex vivo* studies, investigators have also observed reduced MHC expression and T cell infiltration in a mouse lymphoma model expressing mutant EZH2^Y641^. Moreover, an EZH2 inhibitor was found to restore MHC expression in a human DLBCL cell line with an EZH2 mutation. Dersh et al. (2021) used genome-wide CRISPR technology in DLBCL cell lines to demonstrate that EZH2 is the most critical regulator of MHC-I. The present study showed that EZH2 expression was upregulated in subtype C2 and was significantly negatively correlated with MHC-I, and thus, we hypothesised that GOF mutations in EZH2 are another major cause of MHC-I expression downregulation in subtype C2. Based on this theory, drugs acting on EZH2 mutations have been approved for use in a variety of malignancies. For example, tazemetostat (an EZH2 inhibitor) has been used to treat r/r follicular lymphoma patients with EZH2 mutations, achieving an objective response rate of 35% (19/54) (Morschhauser et al., 2020).

Reduced immune infiltration in subtype C2 manifests primarily as T cell exclusion. Both T cell reductions and MHC-I downregulation attenuate CD8^+^ T cell-mediated antitumour immune responses, which might be the primary reason for the worse prognosis associated with subtype C2. Low immune infiltration suggests a worse prognosis, which has been previously demonstrated in most malignancies (Bruni et al., 2020).

In our study, subtype C1 was enriched in immune pathways such as T cell receptors, cytokines, chemokines, and lysosomes, which is consistent with the immune profile of subtype C1. Moreover, we found that the expression of immune checkpoint genes was upregulated in subtype C1, which is consistent with previous research (Autio et al., 2022). T cells express immunosuppressive molecules (e.g., PD-1 and CTLA4) on their surface, which is a sign of T cell “exhaustion”, presenting as T cell dysfunction. This has been confirmed in chronic viral infections and tumours (Wherry et al., 2007; Ahmadzadeh et al., 2009). Therefore, we believe that the T cell activation and exhaustion states are in a dynamic balance in subtype C1. According to the “TIDE” model theory, the expression of co-receptors and costimulatory receptors is strictly regulated by different signals involved in T cell activation and differentiation, and levels of inhibitory receptors are upregulated to counteract co-stimulatory signals after peak stimulation (Zhu et al., 2011). The immune microenvironment induces an inflammatory response while limiting damage to surrounding tissues as an intrinsic protective mechanism. However, patient T cells are chronically stimulated by tumour-specific antigens, and their surface expression levels of inhibitory molecules are continuously increased, leading to impaired effector functions that promote tumour growth (Philip and Schietinger, 2022), which could contribute to the development of subtype C1 tumours. PD-1 antibodies restore the function of exhausted T cells by blocking the inhibitory effect of PD-1/PD-L1 (van der Leun et al., 2020).

Tumour progression is not necessarily due to a single factor. In the present study, subtype C1 was enriched in the TLR signalling pathway, suggesting that this might be relevant to the development of DLBCL. Myeloid differentiation primary response protein 88 (MYD88) is a key adapter molecule in the TLR signalling pathway, transmitting signals from the TLR and interleukin receptor to downstream nuclear factor-κB (NF-κB) and promoting B cell proliferation. MYD88^L265P^ (leucine changed to proline at position 265) is a GOF mutation that results in TLR/AKT/NF-κB pathway activation, and it is found in 29% of patients with activated B-cell DLBCL (Ngo et al., 2011). Interestingly, we found that MYD88 expression levels were significantly elevated in subtype C1 (*p* < 0.05; Supplementary Figure S7), and thus, we believe that MYD88^L265P^ might contribute to the activation of the TLR signalling pathway in subtype C1 and induce tumour development. In addition, activated NF-κB could eventually upregulate PD-L1 expression by activating the JAK/STAT3 signalling pathway (Song et al., 2019). Fu et al. (2021) found a PD-L1 positivity rate of 50% (7/14) in tumour cells of a MYD88^L265P^ group compared to 18.4% in a non-MYD88^L265P^ group of patients with DLBCL (9/49), which implies the possibility that combination therapy can be used.

There are significantly different immunological and genetic characteristics among different immune subtypes of DLBCL. Distinguishing between immune subtypes has important implications for assessing prognosis and developing treatment plans. However, the commonly used methods for tumour immune infiltration assessments are based primarily on gene expression profiles or sequencing data, which are difficult to use widely in clinical practice. Therefore, we aimed to develop a simple and effective assessment tool.

CD8^+^ T cells are the primary component of TILs and the main cells exerting anti-tumour effects. The content of CD8^+^ T cells directly reflects the level of immune infiltration. We obtained 10 hub genes that were highly correlated with CD8^+^ T cells. GO and KEGG enrichment analyses showed that they were primarily involved in T cell activation and immune regulation. Dimensionality reduction was also performed on the 10 hub genes, and the IRGS was obtained for each patient through factor analysis. Correlation analysis verified that both hub genes and the IRGS were significantly correlated with the immune microenvironment. The AUC also showed that the IRGS could be used to accurately differentiate between immune subtypes. Therefore, the IRGS generated based on the 10 hub genes can be used as an alternative scheme to differentiate between immune subtypes. We further classified patients into high- and low-IRGS groups. There was a large degree of immune infiltration, and immunosuppressive molecules were upregulated in the high-IRGS group, which was representative of subtype C1, with T cells in a dysfunctional state. Whereas the low-IRGS group was opposite to the high-IRGS group, which was representative of subtype C2, with T cells showing exclusion. The present study showed that the high-IRGS group was more sensitive to PD-1 antibodies, which was consistent with previous studies. Patients with PD-1^high^CD8^+^ T cells were found to have more efficacious ICBT (Macek Jilkova et al., 2019). Patients with PD-L1^+^/TIL^+^ tumours were more likely to benefit from ICBT (Zhang and Chen, 2016).

Recent studies have demonstrated that the quantification of circulating immune cells, including ALC, can be used to predict tumour outcome and may be considered as surrogate markers of the immune TME (Laddaga et al., 2022). Our retrospective study of 30 patients with r/rDLBCL showed that ALC was higher in patients who responded to combination therapy with PD-1 inhibitors. This suggests that ALC is a good predictive marker for ICBT treatment, which is also observed in other tumours (Valero et al., 2021). Moreover, TME of DLBCL with PD-L1 up-regulation is accompanied by substantial T-lymphocyte infiltration, and such patients are more responsive to PD-1 inhibitors, as observed in patients with r/rDLBCL (Godfrey et al., 2018). These previous studies further support the hypothesis that DLBCL is a tumour with a different inflammatory environment, and that differentiation of subtypes is of great value in guiding ICBT.

Finally, we also analysed the relationship between hub genes and prognosis. Except for that of *CD8A* and *CD3E*, high expression of the remaining hub genes predicted a better prognosis, which is consistent with the better prognosis associated with subtype C1. Genes highly correlated with prognosis were screened using LASSO regression to construct risk models, and survival analysis showed better prognosis for the low-risk group. The discovery of these immune genes, in addition to adding new prognostic assessment metrics, might play a larger role in differentiating immune subtypes based on gene expression levels and predicting the efficacy of immunotherapy. These results also emphasise the importance of the hub genes in the immune microenvironment.

Our study still has some limitations. First, non-immune pathways associated with subtype C2 were not enriched in GSEA, which limited our analysis of tumour-associated pathways in subtype C2. In addition, the interactions between immune gene sets were not included in this study. Finally, we did not retrieve a dataset containing sufficient samples with gene expression and mutation information to validate the mechanisms underlying the regulatory effects of gene mutations on the immune microenvironment, and the interrelationship between the two should be the focus of future studies.

In summary, immune gene set-based immunophenotyping of DLBCL clearly suggests the heterogeneity of different DLBCL immune microenvironments, reflecting the sensitivity of immunophenotyping. The present study also reveals that the development of DLBCL is strongly influenced by the immune microenvironment. An in-depth study of the immune microenvironment could lead to improved clinical decision-making strategies for DLBCL and other tumours.

## Data Availability

The datasets presented in this study can be found in online repositories. The names of the repository/repositories and accession number(s) can be found in the article/supplementary material.
