# Supplementary figures and images for: Gene set-based identification of two immune subtypes of diffuse large B cell lymphoma for guiding immune checkpoint blocking therapy
Source: Front Genet. 2022 Oct 7;13:1000460. doi: 10.3389/fgene.2022.1000460 (PMC9585251; doi:10.3389/fgene.2022.1000460)

Subtype C1 C2

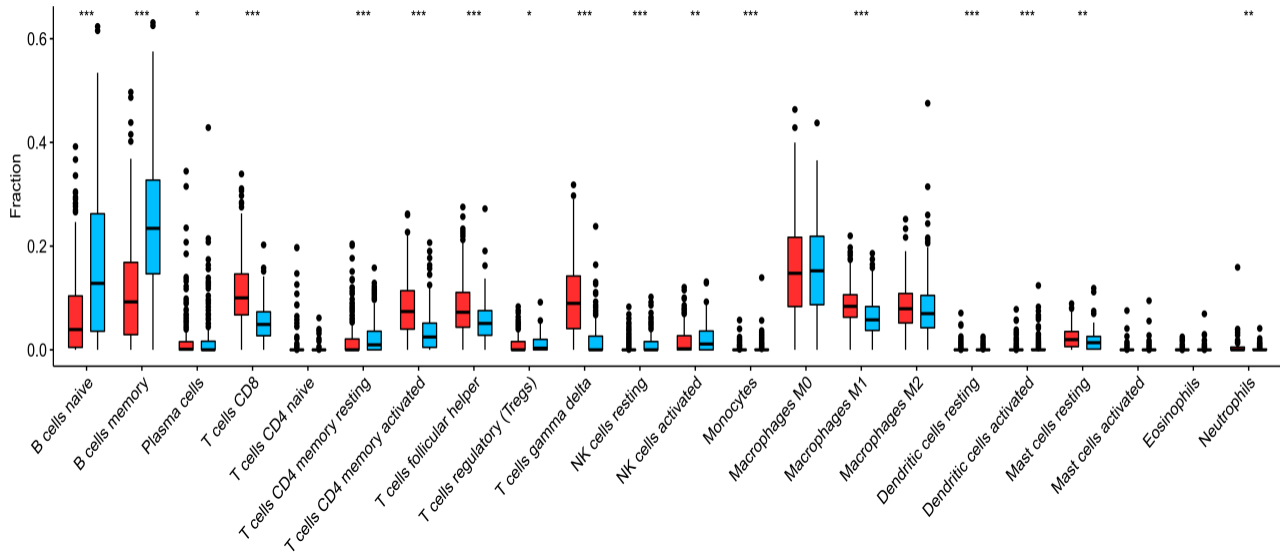

Supplement: Supplementary file 1 [file DataSheet7.PDF]

Type DLBCL normal

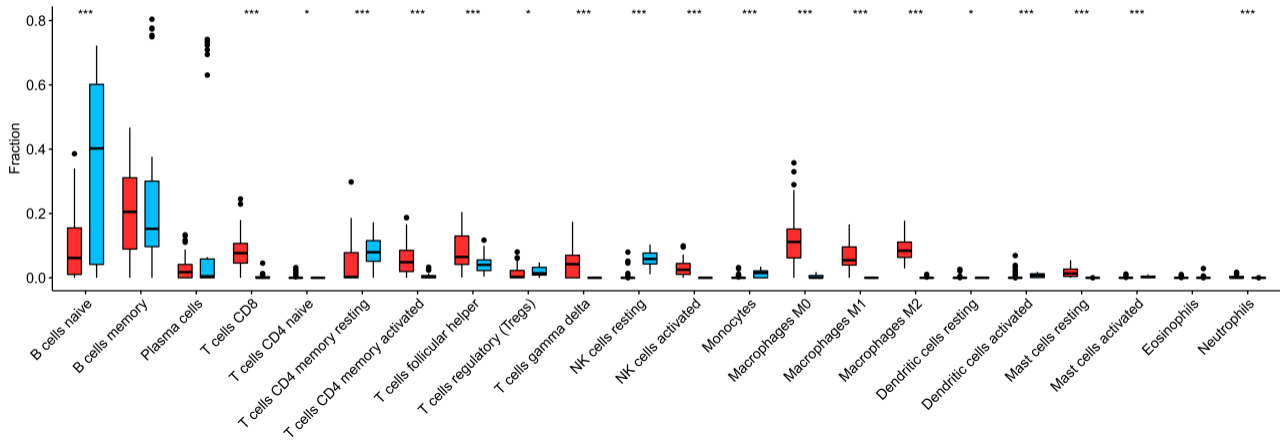

Supplement: Supplementary file 2 [file DataSheet2.PDF]

**A****GSE10846**Subtype 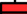 C1 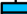 C2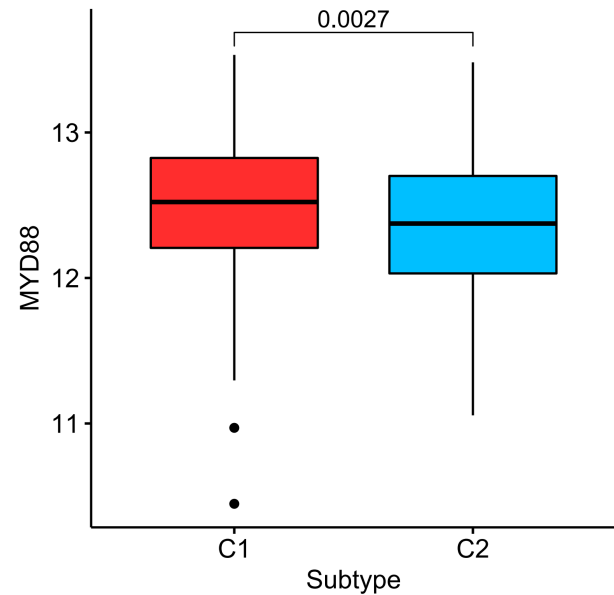**B****GSE32918**Subtype 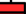 C1 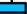 C2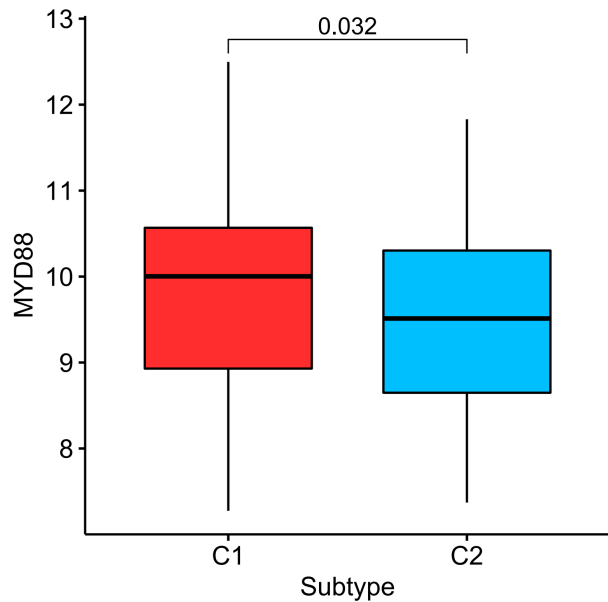

Supplement: Supplementary file 5 [file DataSheet4.PDF]

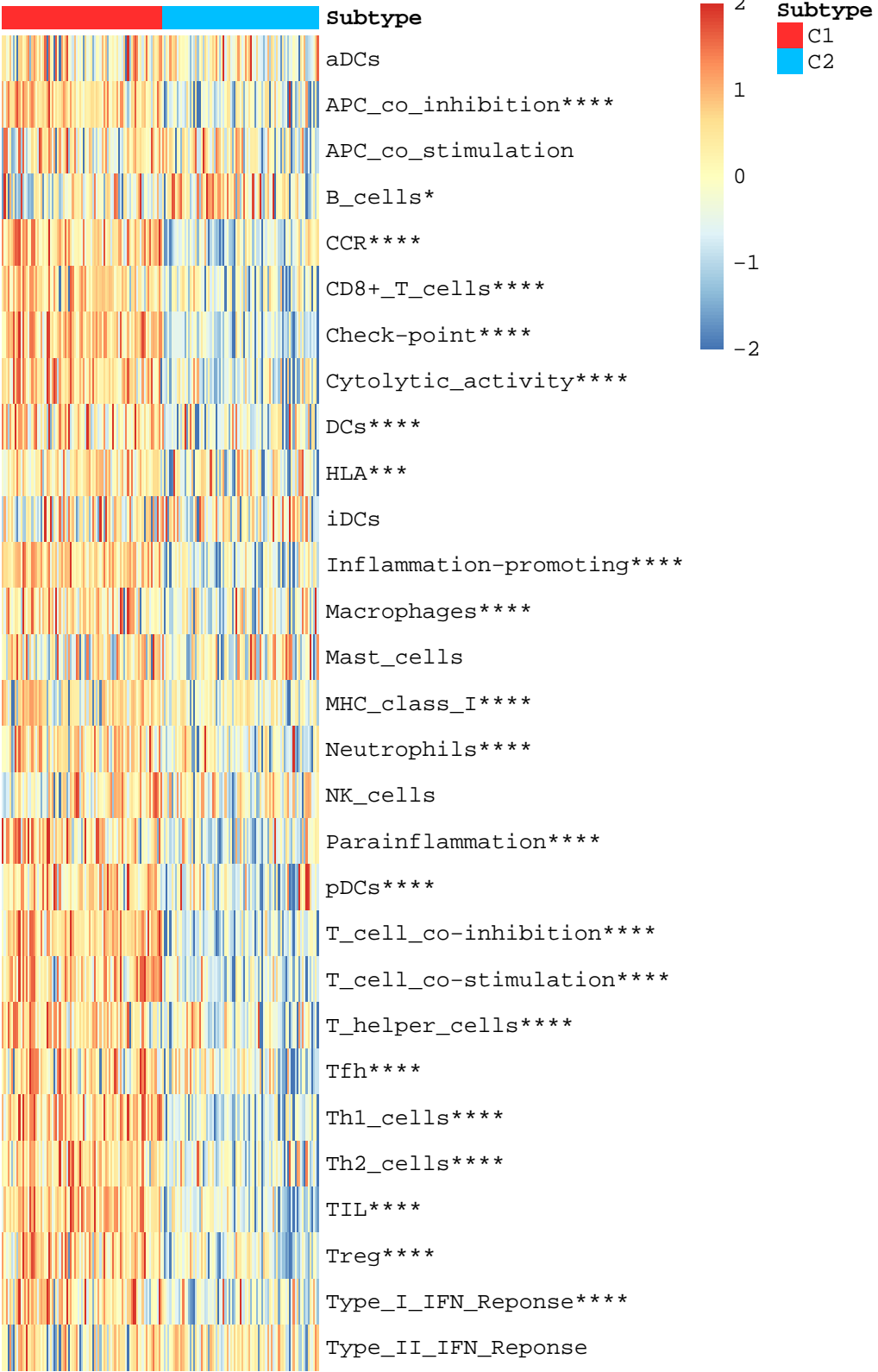

Supplement: Supplementary file 6 [file DataSheet6.PDF]

Type DLBCL normal

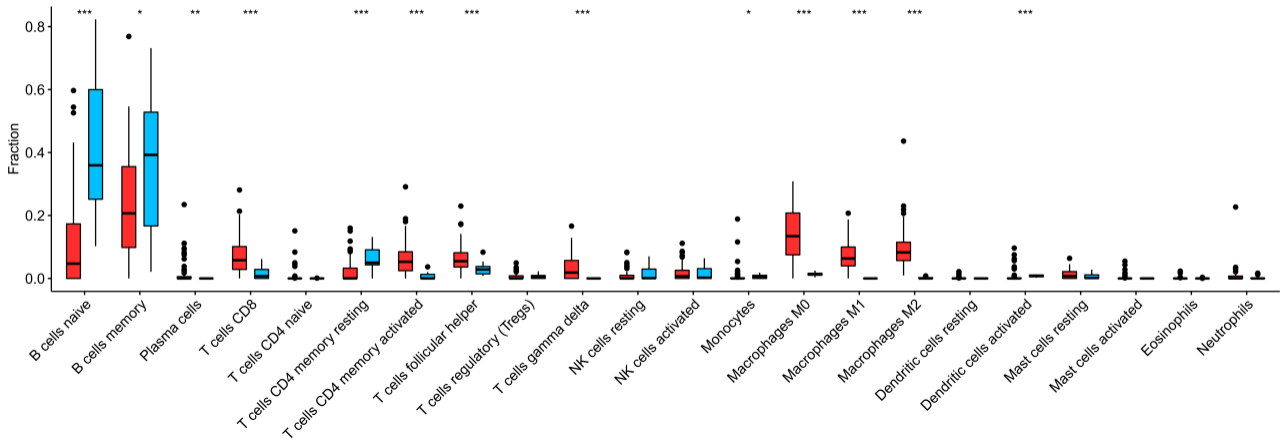

Supplement: Supplementary file 10 [file DataSheet3.PDF]

Subtype ■ C1 ■ C2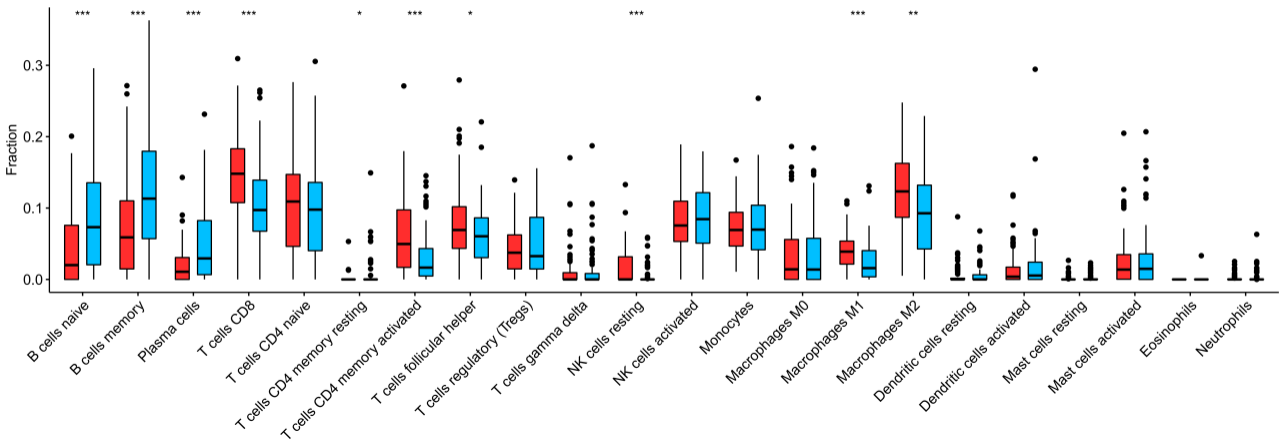

Supplement: Supplementary file 12 [file DataSheet1.PDF]

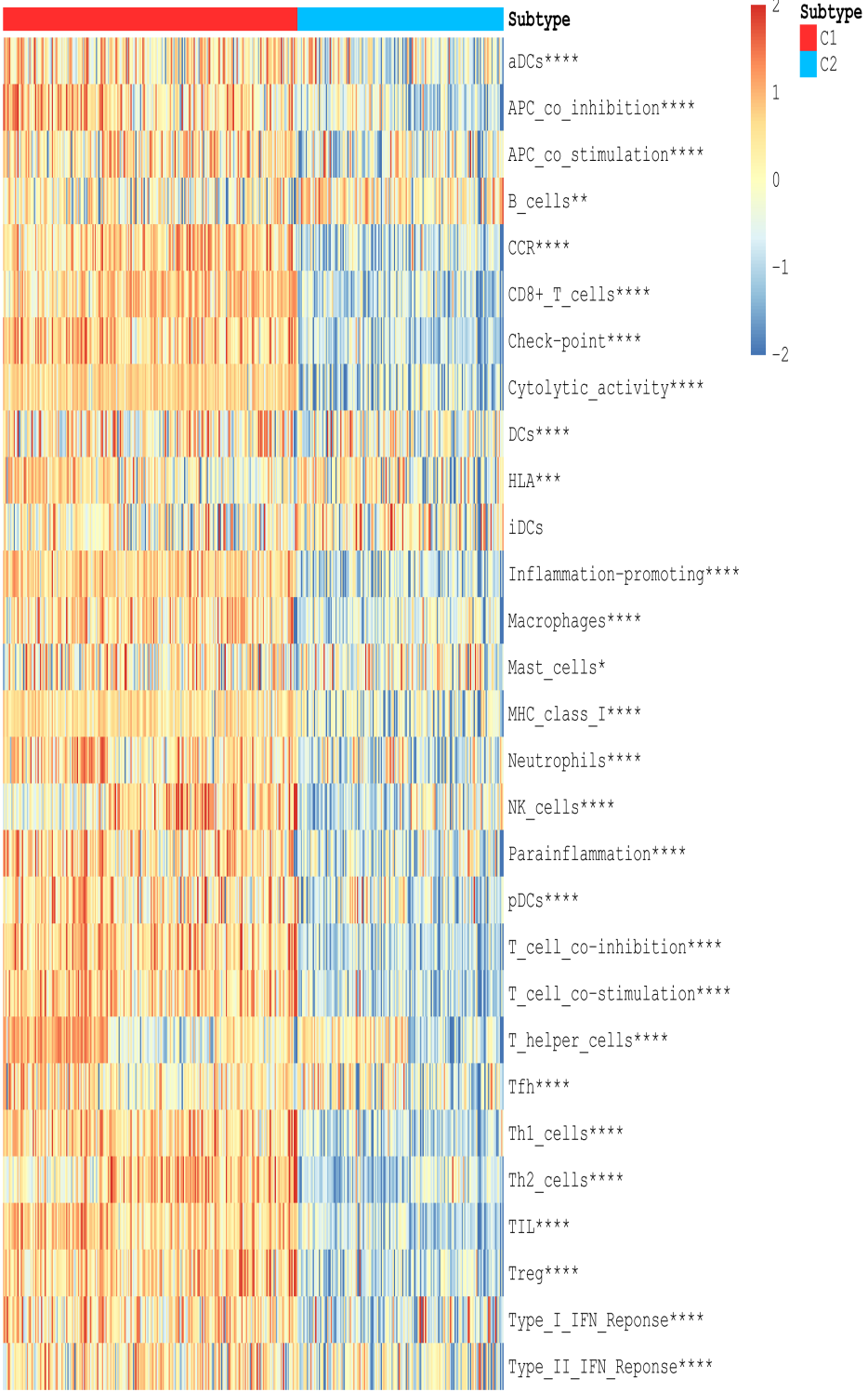

Supplement: Supplementary file 13 [file DataSheet5.PDF]
